# Cycloserine-Induced Psychosis: A Case Report

**DOI:** 10.7759/cureus.77078

**Published:** 2025-01-07

**Authors:** Shrinivas R Raikar, Sreeraj G, Sneha Sneha, Janarthanan Rajkumar

**Affiliations:** 1 Pharmacology, BLDE(DU) Shri B M Patil Medical College, Hospital and Research Centre, Vijayapura, IND

**Keywords:** adverse drug reactions (adrs), antitubercular drug, cycloserine, drug-induced psychosis, multidrug-resistant tuberculosis (mdr-tb), naranjo algorithm

## Abstract

Tuberculosis (TB) is a highly infectious disease and a global health challenge caused by *Mycobacterium tuberculosis*. The disease presents as drug-sensitive TB or drug-resistant TB (DR-TB). DR-TB could be of various types like isoniazid mono-resistant, multidrug-resistant (MDR)-rifampicin mono-resistant (MDR-RR), MDR-TB, extensively DR-TB (XDR-TB), or pre-XDR-TB. Management of DR-TB is challenging due to the longer treatment duration and adverse drug reactions (ADRs) to the second-line anti-TB drugs. Some of these could be life-threatening and require urgent care.

Neuropsychiatric ADRs associated with cycloserine require greater attention due to their potential to cause treatment failure. The objective of this case report is to emphasize the importance of awareness about psychiatric ADRs caused by antitubercular agents. It also highlights the reversible nature of these adverse events upon drug withdrawal. To ensure methodological rigor in the assessment of psychosis, the Brief Psychiatric Rating Scale (BPRS) and Naranjo ADR Probability Scale were employed as diagnostic tools to evaluate the severity of psychiatric symptoms and establish the likelihood of an ADR, respectively. It is emphasized here that proper vigilance with immediate management is essential to avoid fatal outcomes. Herein, a case of ADR caused by cycloserine in a pulmonary MDR-TB case as a part of a new modified longer regimen in the Department of Respiratory Medicine at Shri B M Patil Medical College, Hospital and Research Centre, Vijayapura, Karnataka, is presented.

## Introduction

Mental health and physical health are deeply interconnected, especially in the realm of infectious diseases such as tuberculosis (TB). Modern medicine emphasizes the need for a holistic approach to patient care by acknowledging the profound impact of physical illness treatments on mental health.

In the past, mental disorders were predominantly attributed to spiritual forces. However, contemporary research has highlighted the complexity of mental illnesses, revealing biological, psychological, social, and environmental causative factors. *Mycobacterium tuberculosis* is the etiological agent of TB, which remains a chronic respiratory infection with high mortality rates due to a large number of factors including the bacterial burden, socio-economic conditions, and related pathologies, claiming approximately 1.3 million deaths annually worldwide [1]. In the last few decades, there has been a growing issue concerning this infectious disease because of the increasing number of cases resistant to first-line antitubercular drugs like rifampicin and isoniazid. This leads to higher costs for healthcare services and a higher burden of disease [2,3].

Evidence-based guidelines and international medical societies have considered several treatment options, such as robustly effective extended-spectrum antibiotics such as cycloserine (d-4-amino-3-isoxazolidine), as a means of treating TB-resistant individuals [4,5]. *Streptomyces orchidaceous* produces cycloserine (d-4-amino-3-isoxazolidine), a broad-spectrum antibiotic used in multidrug-resistant (MDR)-TB with second-line antitubercular action [6]. This drug is currently used in combination with other antitubercular drugs, like rifampicin, isoniazid, streptomycin, pyrazinamide, and ethambutol. The use of second-line medications, such as cycloserine, to treat drug-resistant TB strains that are resistant to first-line treatments emphasizes how important it is to manage these cases effectively.

Psychiatric symptoms induced by anti-TB medications, especially in the context of MDR-TB therapies, are well documented, encompassing depression, anxiety, and psychosis. Although cycloserine is effective against TB, it is known to have neuropsychiatric side effects, including severe psychosis, as demonstrated in this case. Early detection and timely management of these symptoms, including prompt psychiatric consultation and possible medicine adjustments, are critical in managing MDR-TB. It is also important to understand the intricate connection between these medications and mental health to achieve optimal treatment outcomes and improve patient’s quality of life.

Neuropsychiatric adverse drug reactions (ADRs) of cycloserine, typically emerging within the first two weeks of therapy and resolving upon discontinuation, include somnolence, headache, tremor, confusion, irritability, psychosis, suicidal thoughts, and seizures. In individuals with epilepsy, cycloserine is contraindicated and should be used with caution in those with a history of depression because of an increased risk of suicide. Cycloserine has also been reported to exacerbate symptoms in chronic schizophrenia and can induce delirium [7].

Particularly in the treatment of MDR-TB, recent research emphasizes the significance of psychiatric assessments both prior to and during cycloserine medication. These assessments can significantly enhance the early detection and management of potential neuropsychiatric ADRs, particularly in patients receiving drugs like cycloserine. Treatment programs for MDR-TB must also include psychological support services to address side effects quickly. The decision to highlight cycloserine is based on its known mechanism of action involving N-methyl-D-aspartate (NMDA) receptor antagonism, which has been linked to neuropsychiatric side effects. To minimize the risk of severe neuropsychiatric episodes, updated guidelines now advocate for routine monitoring of mental health symptoms and the adjustment of treatment plans accordingly. This approach not only enhances treatment efficacy but also ensures comprehensive care for the patient. Furthermore, this research emphasizes the importance of tailoring treatment plans to the individual's mental health history to prevent serious adverse reactions. The aim of this report is to highlight a case of cycloserine-induced psychosis in a 26-year-old female patient on treatment for MDR-TB at Shri B M Patil Medical College, Hospital and Research Centre, Vijayapura, Karnataka.

## Case presentation

Patient history

A 26-year-old female patient was initially diagnosed with TB in September 2023 at a secondary healthcare facility. On May 29, 2024, she was rediagnosed with MDR-TB. She had previously been on a standard anti-TB treatment regimen (ATT-4) consisting of isoniazid, rifampicin, pyrazinamide, and ethambutol for three months, followed by ATT-2 (isoniazid and rifampicin) for six months. After this, she developed a cough with expectoration, which was mucoid, non-blood-tinged, and non-foul-smelling, which occurred more in the early morning. It was aggravated while lying in the left lateral decubitus position. Consequently, she was referred to the Department of Respiratory Medicine, Shri B M Patil Medical College, Hospital and Research Centre, Vijayapura, Karnataka, where she was admitted.

Diagnostic findings

High-resolution computed tomography (HRCT) was done. The CT report revealed multiple central lobular nodules, some of them showing a “tree in bud appearance” involving the right upper and middle lobes. Multiple consolidated patches with breakdown cavitations in the upper and middle lobes in the right lung fields predominantly in the apical segment of the right upper lobe suggest an active infective etiology (Koch’s etiology). There is a complete collapse of the left lung with significant volume loss, architectural distortion, cystic bronchiectatic changes, and mediastinal shift toward the left side indicating sequelae of old granulomatous etiology.

In the cartridge-based nucleic acid amplification test (CB-NAAT), *M. tuberculosis*, which was resistant to the drug rifampicin, was detected. The fluoroquinolone line probe assay (FLLPA) test detected *M. tuberculosis* and resistance to rifampicin, and in the single-locus line probe assay (SLLPA), *M. tuberculosis*, which was resistant to levofloxacin and moxifloxacin but not to streptomycin, were detected.

HRCT and CB-NAAT were chosen based on their high sensitivity and specificity for detecting active TB and confirming drug resistance. These diagnostic methods were used to guide appropriate treatment decisions.

Treatment initiation

The patient was initiated on a modified longer regimen comprising second-line ATT: Tab. bedaquiline 200 mg once in two days in the morning, Tab. delamanid 100 mg (BD), Tab. linezolid 600 mg (OD), Tab. cycloserine 500mg (OD), Tab. clofazimine 100 mg (HS) at night, Tab. pyridoxine 100 mg (HS) at night, Tab. sildenafil 20 mg (TID), Tab. ferrous ascorbate and folic acid (OD), and Tab. pantoprazole 40 mg (OD). Although other second-line anti-TB drugs were considered, cycloserine was chosen for its effectiveness against resistant strains and its suitability for the patient's overall clinical condition.

Development of ADRs

After 10 days of commencing the modified longer regimen, the patient exhibited behavioral changes including reduced interaction, irritability, irrational talk, aggression, reduced sleep, refusal to eat, visual hallucinations, restlessness, and unusual claims. The patient had no prior psychiatric history, did not use any psychoactive substances, and had no family history of psychiatric or other medical conditions. Additionally, no psychiatric evaluation was conducted before initiating the MDR-TB regimen.

Upon examination, her body weight was 35 kg. Routine investigations, including a full blood count with differentials, electrolyte levels, urea, creatinine, urinalysis, and thyroid function tests, were all within normal ranges except for the hemoglobin level of 9.2 g/dL (anemic).

Intervention and outcome

A psychiatric consultation revealed increased psychomotor activity, incoherent speech, visual hallucinations, and delusions. Physical examination showed mild pallor, weight loss, and generalized lymphadenopathy.

Using a seven-point scoring system, the Brief Psychiatric Rating Scale (BPRS) evaluates 18 items to determine the severity of mental disorders, including psychosis [8]. Severe symptoms are indicated by higher ratings, and a notable improvement in scores indicates that treatment is working. To determine the probability that a particular prescription may produce an ADR, a standardized method called the Naranjo ADR Probability Scale [9] is utilized. The ability to objectively evaluate the causal association between drug exposure and mental symptoms makes it important for identifying drug-induced psychosis; higher scores (≥9) indicate a higher risk of drug-induced psychosis. A summary of clinical manifestations, diagnostic assessments, interventions, and therapeutic outcomes is shown in Table 1.

**Table 1:** Summary of clinical manifestations, diagnostic assessments, interventions, and therapeutic outcomes ADR: adverse drug reaction; BPRS: Brief Psychiatric Rating Scale

Parameter	Description	Findings/results
Clinical manifestations	Psychiatric symptoms including irritability, aggression, visual hallucinations, and sleep disturbances	Onset: 10 days after initiating Tab. cycloserine. Symptoms: aggression, hallucinations, irritability, restlessness, reduced sleep
Diagnostic assessments	Use of BPRS and Naranjo ADR Scale to assess severity and causality of psychiatric symptoms	BPRS score: 50 (severe), Naranjo ADR score: 7 (probable)
Interventions administered	Tab. cycloserine discontinued; antipsychotic (Tab. olanzapine) and Tab. vitamin B complex prescribed	Tab. olanzapine, dose: 10 mg once daily for 10 days and Tab. vitamin B complex: twice daily for 15 days
Therapeutic outcomes	Improvement in psychiatric symptoms following intervention	BPRS score: reduced to 20 (within 72 hours). Symptom improvement: significant improvement within 3 days

The BPRS score was 50, and the Naranjo algorithm score was 7, in this case suggesting probable drug-induced psychosis. Cycloserine was identified as the likely cause, and it was discontinued while the remaining anti-TB drugs were continued without modification. The patient was prescribed a low dose of antipsychotic medication (Tab. olanzapine 10 mg at night for 10 days) and Tab. vitamin B complex twice daily for 15 days. Counseling was also provided to her family. Within 72 hours, the BPRS score reduced from 50 to 20, indicating significant improvement in psychiatric symptoms. Cycloserine-induced psychosis was considered the probable diagnosis. The timeline of events is depicted in Table 2.

**Table 2:** Timeline of events: development, diagnosis, and management of cycloserine-induced psychosis in a multidrug-resistant tuberculosis patient

Date	Event
September 15, 2023	The patient was initially diagnosed with tuberculosis (TB) at a secondary healthcare facility
May 29, 2024	Rediagnosed with multidrug-resistant tuberculosis (MDR-TB) following symptoms indicative of treatment failure
May 30, 2024	Started on modified longer regimen, including second-line drugs such as Tab. cycloserine, Tab. bedaquiline, and Tab. delamanid
June 09, 2024	The onset of psychiatric symptoms: behavioral changes, visual hallucinations, irritability, and aggression
June 20, 2024	Cycloserine was discontinued; antipsychotic therapy (Tab. olanzapine) was initiated; significant symptom improvement was seen within 72 hours

## Discussion

Cycloserine, a broad-spectrum antibiotic derived from *S. orchidaceous*, was initially isolated in the year 1954 and subsequently synthesized [10]. When first-line drugs such as isoniazid, rifampin, ethambutol, pyrazinamide, and streptomycin are ineffective because of resistance, it is used in combination with other antitubercular medicines to treat pulmonary or extrapulmonary TB.

Second-line antitubercular drugs have a well-established history of ADR especially when treating DR-TB. These reactions can lead to severe or even fatal outcomes. Though this might be challenging in settings with a large patient volume, effective care frequently entails routine outpatient follow-ups and counseling.

The development of psychosis has been associated with a number of medications, such as fluoroquinolones, cycloserine, ethambutol, and isoniazid. Among these, cycloserine is especially well known for its capacity to cause ADRs linked to the central nervous system (CNS) and mental health [11]. In this particular case, detailed clinical and diagnostic evaluations revealed that psychosis was the adverse reaction to cycloserine. Cycloserine was probably the cause of psychosis, as it subsided after the drug was halted. Since the symptoms did not return after commencing other medications, follow-up monitoring confirmed that no more antitubercular medications were involved. Delayed recognition of these serious adverse events may result in life-threatening outcomes, including suicide, as documented in the literature.

It is not clear how cycloserine causes psychosis. However, according to certain research, cycloserine may function at the glycine sites of NMDA receptors as a partial agonist or antagonist. The potential neuropsychiatric effects of cycloserine are largely attributed to its antagonism on NMDA receptors, which has been well documented in several studies [12]. At elevated doses, it may operate as an NMDA antagonist, potentially aggravating or causing psychotic symptoms [13]. Previous investigations, including those by Sharma et al. [14], have shown different signs of cycloserine-induced psychosis, including aggressive behavior and hepatic dysfunction.

Although cycloserine is an essential part of DR-TB therapy plans, it carries a high risk of ADRs. Psychiatric assessments are therefore necessary before starting this medicine. Even though national guidelines advise such assessments before beginning DR-TB therapy, this case shows that cycloserine-induced psychosis can still occur even with prior assessment. This highlights the significance of continuous psychiatric monitoring throughout the entire course of MDR-TB treatment and not just during the initiation phase. The regular assessments allow for the early detection of neuropsychiatric ADRs and facilitate the prompt management of any developing symptoms.

Cycloserine inhibits *M. tuberculosis* at doses between 5 and 20 µg/mL in vitro, and no cross-resistance with other antitubercular drugs was seen. In three to four hours after oral dosing, 70%-90% of cycloserine reaches peak plasma concentrations due to its rapid absorption. Concentrations in cerebrospinal fluid (CSF) are similar to those in plasma, and it is extensively dispersed throughout bodily fluids and tissues. Within the first 12 hours, almost half of the dosage is eliminated unchanged in the urine with minimal metabolic processing [15]. These pharmacokinetic properties may contribute to the rapid onset and manifestation of cycloserine-related adverse events. The optimal dosing of cycloserine is unknown [16], but modeling studies suggest doses from 250 to 750 mg twice daily, with 500 mg twice daily for paucibacillary disease and 750 mg twice daily for cavitary pulmonary disease [17].

Cycloserine-induced CNS reactions usually appear in the first two weeks of treatment and subside after discontinuation. A few of the symptoms include paranoia, visual hallucinations, psychotic states, disorientation, impatience, and less commonly seizures. In this case, the patient had symptoms such as behavioral changes, including reduced interaction, irrational talk, irritability, aggression, reduced sleep, refusal to eat, visual hallucinations, and restlessness. The temporal relationship between the onset of symptoms and the administration of cycloserine, as well as the remission of symptoms upon termination of the prescription, provide strong evidence for the attribution of these symptoms to cycloserine.

The strength of this case is the thorough evaluation of the patient's clinical history, diagnostic work-up, and the clear temporal association between cycloserine administration and the emergence of neuropsychiatric symptoms, which collectively strengthen the likelihood that cycloserine contributed to these effects. However, it is important to recognize the limitations of our findings, as reflected in a Naranjo score of 7, indicating a probable but not conclusive causal relationship.

This case was reported to the Adverse Drug Monitoring Centre PvPI, BLDE(DU) Shri B M Patil Medical College, Hospital and Research Centre, Vijayapura, Karnataka. The worldwide unique ID for this case is IN-IPC-300916686 under the Indian Pharmacopoeia Commission.

## Conclusions

This case emphasizes the need for routine psychiatric assessments during MDR-TB treatment to prevent severe adverse events and improve patient outcomes. This also underscores the importance of prompt vigilance, which should be taken while administering cycloserine in MDR-TB patients because of the potential for causing psychiatric-related adverse events.

There is a need for further pharmacovigilance studies to better understand the mechanism and management strategies for cycloserine-induced psychosis. Enhanced data collection and analysis will be crucial in informing treatment guidelines and improving patient outcomes in the future. Healthcare providers should incorporate specific psychiatric screening tools such as the BPRS when managing patients receiving second-line anti-TB drugs, including cycloserine. Furthermore, integrating mental health services into TB treatment programs should be considered as a standard practice to monitor and manage neuropsychiatric ADRs effectively. This will not only help prevent the precipitation of ADRs but also guarantee optimal care to the patients.
